# Hypothetical Pathway for Formation of Cholesterol Microcrystals Initiating the Atherosclerotic Process

**DOI:** 10.1007/s12013-020-00925-2

**Published:** 2020-06-30

**Authors:** Witold K. Subczynski, Marta Pasenkiewicz-Gierula

**Affiliations:** 1 Department of Biophysics, Medical College on Wisconsin, 8701 Watertown Plank Road, Milwaukee, WI 53226 USA; 2 Department of Computational Biophysics and Bioinformatics, Jagiellonian University, ul. Gronostajowa 7, 30-387 Krakow, Poland

**Keywords:** Atherosclerosis, Cholesterol, Cholesterol domains, Cholesterol crystals, Membrane

## Abstract

Major factors leading to the development of atherosclerosis are a high cholesterol (Chol) level in the blood and oxidative stress. Both promote the formation of Chol microcrystals in blood vessel walls. Deposition of Chol microcrystals in arterial intima causes inflammation, which initiates and accompanies the atherosclerotic process in all its phases. One of the possible sources of Chol in the blood vessel walls is oxidized low-density lipoproteins—this atherosclerotic plaque formation pathway has already been described in the literature. Here, we hypothesize that initiation of the atherosclerotic process may involve Chol domains in the plasma membranes of arterial cells. Increased Chol content and the presence of polyunsaturated phospholipids in these membranes together with oxidative stress (phospholipid peroxidation) may lead to the formation of pure Chol bilayer domains that, with further peroxidation and increased Chol content, may collapse in the form of Chol seed crystals. Independent of their origin, Chol microcrystals activate inflammasomes, thereby stimulate immune responses, and initiate inflammation that may lead to the development of atherosclerosis. This new, hypothetical pathway has not yet been investigated in depth; however, data from the literature and our own results support its feasibility.

## Introduction

Biological and clinical research indicates a fundamental and initiating role of inflammation in atherosclerosis [1, 2]. Inflammation is the body’s immune system response to harmful stimuli. Among such stimuli are endogenous crystalline substances, and cholesterol (Chol) microcrystals are shown to be one of them [3]. The presence of extracellular macroscopic monohydrate Chol crystals in advanced atherosclerotic lesions was demonstrated in the 1970s [4]. However, these crystals are not responsible for early inflammation; rather, they are an effect of atherosclerosis progression and play a role in atherosclerotic plaque rupture [5–7]. Currently, the accepted view is that cardiovascular inflammation is prompted by Chol microcrystals at the initiation of the atherosclerotic process.

Duewell et al. [8] and Rajamäki et al. [9] established a direct correlation between Chol microcrystals, inflammation, and atherosclerosis at the initial state of atherosclerosis. In experiments on germ-free mice fed on a high-cholesterol diet leading to atherosclerosis development, Duewell et al. [8] showed that detection of Chol microcrystals in early atherosclerotic lesions coincided with the appearance of inflammatory cells. Also shown was that microscopic Chol crystals injected intraperitoneally into mice under defined conditions induced acute inflammation [8].

Both in vivo and in vitro studies revealed that Chol crystals are phagocytosed by macrophages [3, 9–11]. This leads to lysosomal destabilization and activation of the NLRP3 inflammasome [3, 8, 9], which prompts vascular inflammation [8–10]. However, it was also demonstrated in vitro that free Chol crystalized intracellularly in macrophages [12, 13]. At present, it is not feasible to establish that the same process takes place in vivo [11], but it is likely that it does [8].

Here, we would like to discuss the plausible sources of free Chol necessary for the formation of Chol microcrystals. The presently accepted opinion is that free Chol is obtained from oxidized low-density lipoproteins (oxLDL) or other modified lipoprotein particles taken up by monocyte-derived macrophages in the arterial intima, which transforms them into macrophage foam cells [14, 15]. An excess of free Chol present in foam cells leads to Chol crystal nucleation. In vitro studies showed that small Chol crystals appeared as early as 1 h after incubation of macrophages with oxLDL and that these crystals increased in size with increasing incubation time [8]. Despite the large body of experimental evidence, Libby et al. [14, 16] is skeptical about the LDL oxidation hypothesis in relation to human atherosclerosis. In this paper, we propose a different hypothetical pathway leading to Chol crystal nucleation in the blood vessel wall.

## A New Hypothetical Pathway Leading to the Formation of Cholesterol Microcrystals

We hypothesize that the process of a Chol crystal nucleus formation is initiated in membranes containing a large amount of Chol. When the amount of Chol in a membrane exceeds the saturation limit, Chol bilayer domains (CBD) form in the membrane [17]. A further increase of Chol content above the Chol solubility threshold induces formation of Chol crystals [17–20]. The peroxidation of phospholipids in a membrane containing a large amount of Chol does not change the relative proportion of Chol and phospholipids; however, it lowers the amount of Chol needed for CBDs to start to form [21–23]. Thus, conditions driving the development of atherosclerosis (high-Chol level and oxidative stress) lead to the situation wherein a membrane oversaturated with Chol is no longer able to accommodate some or all of the CBDs, so the CBDs detach from the membrane and collapse (i.e., fall out of the membrane either inside or outside of the cell) in the form of Chol aggregates that can become crystal nuclei and, in time, convert into Chol monohydrate crystals. As demonstrated experimentally [3, 10], tiny Chol aggregates can be easily phagocytosed by macrophages, which eventually activates inflammasomes [9, 24, 25] and triggers inflammation. After phagocytosis, macrophages transform into foam cells [10], where the Chol crystal nuclei can continue the nucleation process to become monohydrate crystals. Thus, independent of the pathway of Chol microcrystals formation in vascular wall, the crystals can activate inflammasomes and induce inflammation, which may lead to the development of atherosclerosis. In the first of the two pathways discussed, the key role is played by oxLDL [26, 27]; in the second, the key role is assigned to membrane CBDs, which are the precursors of Chol crystals [17, 18]. The following scheme illustrates these two parallel processes:
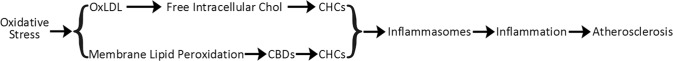


where CHC is Chol crystal.

## Evaluation of the Hypothesis

### Formation of CBDs Precedes Formation of Chol Crystals

CBDs are pure Chol domains of a liquid-ordered phospholipid-Chol bilayer saturated with Chol (1:1 molar ratio). They can form in the phospholipid-Chol bilayer oversaturated with Chol. The phase of such a system is classified as a structured liquid-ordered [28] or dispersed [29]. When the Chol content in the phospholipid bilayer further increases and exceeds the Chol solubility threshold [17, 18, 20, 30, 31], Chol crystals form, presumably outside the membrane [31–33]. Thus, the formation of CBDs precedes the formation of Chol crystals.

### Phospholipid Composition Controls the Chol Content at Which CBDs and Chol Crystals Start to Form

The issue concerning the relationship between the Chol bilayer content and the formation of CBDs (Chol saturation limit) and Chol crystals (Chol solubility threshold) was discussed in detail in ref. [18]. Because phosphatidylcholine (PC), phosphatidylethanolamine (PE), and sphingomyelin (SM) are the major phospholipids of arterial cell membranes, we are interested in the values of their parameters. The established values of the Chol saturation limit are ~33, ~50, and ~50 mol% Chol in the PE, PC, and SM bilayers, respectively, and the established values of the Chol solubility threshold are 50, 66, and 66 mol% Chol in the PE, PC, and SM bilayers, respectively [18]. At values above the Chol solubility threshold, cholesterol crystals form outside these bilayers.

**Fig. 1:**
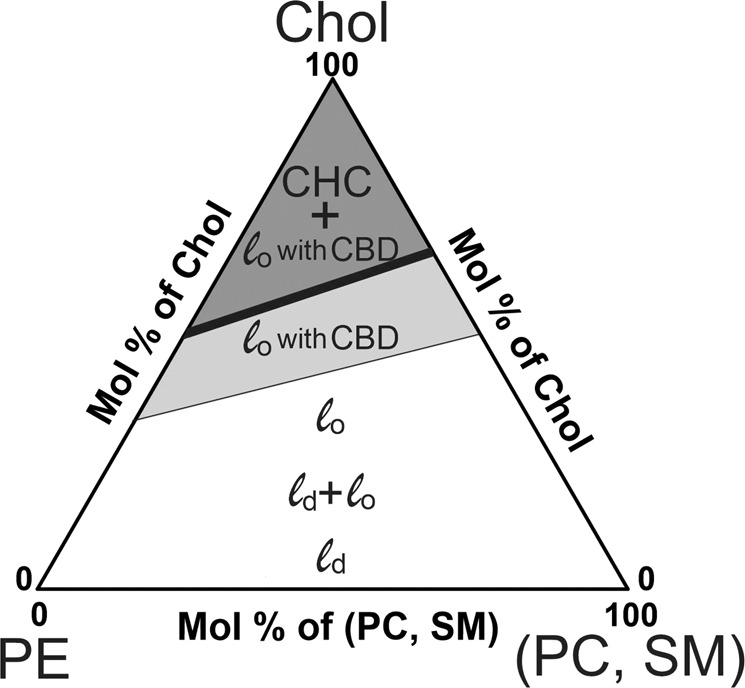
Proposed phase diagram for mixtures of the most abundant phospholipids of the arterial cell membrane (PE, PC, and SM) and Chol. The values of the Chol saturation limit and the Chol solubility threshold are shown by the thin and thick lines, respectively. The white area represents the gradual change of the liquid disordered (*l*_d_) phase, to the coexisting liquid disordered and liquid ordered (*l*_d_ + *l*_o_) phases, and to the liquid ordered (*l*_o_) phase with the increasing Chol content up to the saturation limit (thin line). The light gray area represents the structured liquid ordered phase in which the *l*_o_ bilayer contains CBD (*l*_o_ with CBD); the upper boundary of this phase is the Chol solubility threshold (thick line). The dark gray area represents the structured liquid ordered phase in equilibrium with Chol crystals (CHC) that are a new phase (CHC + *l*_o_ with CBD) (color figure online)

The values of the Chol saturation limit and solubility threshold for a membrane composed of PE, PC, and SM that best represent the lipid composition of the arterial cell membrane can be estimated as the weighted sums of individual values for each phospholipid with the weight equal to the mol% of the lipid in the mixture. Such an estimate enabled us to make the phase diagram shown in Fig. 1. This diagram identifies regions where CBDs are formed within the surrounding phospholipid-Chol bilayer saturated with Chol, and where Chol crystals form outside the bilayer. These Chol crystals constitute a new phase in the system. The phase boundary coinciding with the Chol solubility threshold separates the one-phase region, which is the structured liquid-ordered phospholipid bilayer saturated with Chol and with embedded CBDs (also in the liquid-ordered phase), from the two-phase region where the structured liquid-ordered phase coexists with Chol crystals. Among the main phospholipids constituting the arterial cell membrane, only PE can decrease the values of the Chol content at which CBDs and Chol crystals start to form. Thus, the balance between the phospholipid composition and the Chol content might be considered a new, unexplored mechanism that regulates the Chol-dependent processes in cell membranes.

### Chol and Phospholipid Oxidation as Factors Affecting Chol Saturation Limit and Solubility Threshold

The phase diagram (Fig. 1) was constructed based on the results obtained for phospholipids with both saturated chains or with one saturated and one mono-*cis*-unsaturated acyl chain. Keeping in mind that in naturally occurring unsaturated (singly or poly) phospholipids, predominantly one acyl chain is saturated and the other unsaturated [34]. Phospholipids with polyunsaturated chains decrease the Chol solubility threshold in model membranes [35, 36] as well as the Chol saturation limit [23]. The lowered Chol solubility threshold in *cis*-unsaturated PC-Chol bilayers is due to the reduced mixing of Chol with *cis*-unsaturated acyl chains in the bilayer; in effect, the Chol molecules separate from the phospholipids and form cholesterol-rich domains. The mechanisms behind this effect were analyzed in ref. [37]. It has been also shown that phospholipid peroxidation decreases to a considerable extent the Chol concentrations at which formation of CBDs [21, 22] and Chol crystals [31] are initiated. Peroxidation mainly involves the polyunsaturated chain and introduces a hydroperoxide group into the chain at a double-bond position. Phospholipid hydroperoxide may undergo fragmentation to yield a truncated chain with the terminal aldehyde or carboxylic group [38, 39]. Thus, peroxidation brings a polar group into the nonpolar bilayer core that drives the truncated chain toward the aqueous phase [39, 40]. All this significantly destabilizes the bilayer structure, affects PC-Chol interactions, and leads to a clustering of the Chol molecules in the membrane that, in effect, decreases the Chol solubility threshold and saturation limit.

**Fig. 2:**
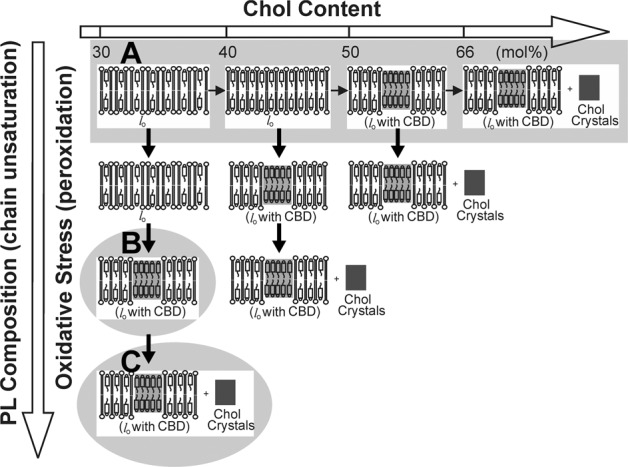
This schematic drawing indicates that the acyl chain unsaturation and oxidative stress (formation of phospholipid peroxides) are the major factors decreasing the Chol content at which CBDs and Chol crystals start to form. In a bilayer made of saturated phospholipids (e.g., modeling the human eye lens membrane), CBDs, and Chol crystals start to form when the Chol content exceeds ~50 and ~66 mol% Chol, respectively (**a**). In a bilayer made of polyunsaturated phospholipids (dilinoleoylphosphatidylcholine), CBDs are observed at ~37 mol% (**b**). Autoxidation of this bilayer suspension induces formation of Chol crystals (**c**). This figure is reproduced from ref. [18], Copyright 2019, with permission from Taylor & Francis

The effects of the Chol content, lipid composition, degree of chain unsaturation, and lipid oxidation state on the bilayer phases are summarized in Fig. 2. This figure can be treated as a universal guideline when investigating Chol-dependent processes in membranes.

## Discussion

Molecular level processes related to atherosclerosis initiation and progression are very complex. Even though they have been studied intensively, they are not fully elucidated. The current view is that the key factor responsible for initiating atherosclerosis is LDL that undergoes oxidation by extracellular matrix proteins in the artery wall. However, clinical trials with antioxidant vitamins or folic acid in patients with preexisting atherosclerosis have proved disappointing [16, 41, 42] or had conflicting results [43]; moreover, direct in vivo evidence for the participation of LDL oxidation in human atherosclerosis remains scarce [16]. Even though the results have been negative or inconclusive, they do not invalidate the oxidation hypothesis of atherosclerosis [42] as oxidation may not concern only LDL.

Here, we propose an alternative source of the artery wall Chol crystals potentially involved in the atherosclerosis development. We hypothesize that these crystals appear in the wall as a consequence of high-Chol content in artery wall cell membranes accompanied by peroxidation of the membrane phospholipids. As indicated in the phase diagram in Fig. 1 and scheme in Fig. 2, in such conditions, CBDs, the potential Chol crystal nuclei in the form of the collapsed CBDs, detach from the membrane. They can be phagocytosed by macrophages where they can undergo further nucleation.

Thus, according to our hypothesis, the molecular events responsible for the onset of atherosclerosis occur in the lipid matrix of the arterial cell membrane where CBDs, the precursors of Chol crystals, are formed. This hypothesis is supported by our recent experimental results [44], which demonstrated that CBDs are present in intact biomembranes with high-Chol content.

Our findings draw attention to the role that membrane lipids may play in controlling the direction of the CBD and Chol crystals formation process and, thus, controlling initiation of atherosclerosis development. Potential endogenic controlling factors include the lipid composition of the membranes of artery wall cells, phospholipid chain unsaturation, and the extent of phospholipid peroxidation. At present, these endogenic factors cannot be utilized for therapeutic purposes, so we can only suggest that all plausible pathways should be considered and discussed in detail to enable better understanding of the complex process of atherosclerosis initiation and progression.

The connection between Chol membrane microdomains and extra-membrane Chol crystals in atherosclerosis development was first identified by Mason and Jacob [31]. They suggested that the membrane microdomains may be a key nucleating site for extracellular cholesterol crystals. However, Mason and Jacob experimentally characterized these microdomains as pure Chol crystalline domains. Such crystal-like Chol domains are, in fact, preparation artifacts and are vastly different from CBDs, which are densely packed, liquid-ordered dynamic structures [45–47]. A concept similar to that of Mason and Jacob [31] was presented by Varsano et al. [48], who demonstrated experimentally that three-dimensional Chol crystals nucleated on the pure Chol domains in the phospholipid-Chol bilayer. Here again, Chol domains were identified as two-dimensional Chol crystalline domains formed within the supported phospholipid-Chol bilayer; to oversaturate the bilayer, the excess Chol molecules were delivered to the bilayer by cyclodextrin. It should be stressed here that our hypothesis concerns the involvement of Chol microcrystals in the initiation of atherosclerosis itself, whereas the study by Varsano et al. [48] concerns the involvement of Chol crystals in the initiation of atherosclerotic lesions.

For most organs and tissues, formation of CBDs and Chol crystals is considered a sign of pathology [31, 49]. However, this is not true for the eye lens, where high-Chol content in the fiber cell membranes and the presence of CBDs is beneficial [50–53]. Chol crystals are detected in lenses extracted from aged human eyes, where the total Chol content in fiber cells is very high (exceeding the Chol solubility threshold) [33, 54, 55]. Chol crystals are not present in lenses from younger donors wherein the relative amount of Chol in eye lenses is smaller [33]. Because oxLDL cannot be the source of the lens Chol (see below), Chol crystals can form entirely through a pathway involving fiber cell plasma membranes and CBDs. Recently, pure CBDs have been detected in the intact membranes of fiber cells [44].

The above-mentioned age-related changes occur in normal lenses [33, 54] and neither pathology nor extensively compromised lens transparency is observed there. Inflammation does not appear to be involved in cataract formation in the eye lens. The lens is an avascular organ and, soon after formation, its fiber cells lose their intracellular organelles [56, 57]. All of these factors protect the lens from the harmful induction of inflammasomes by Chol crystals as well as from initiation of the inflammatory cascade.

The data obtained for the eye lens and the lens fiber cells do not contradict the inflammatory nature of atherosclerosis induced by Chol microcrystals in the artery wall but they do not support the LDL oxidation pathway for formation of Chol microcrystals initiating inflammation. Finally, we want to state that the proposed pathway for formation of Chol microcrystals should be universal to any tissue where high-Chol content in membranes and oxidative stress conditions are present; and depending on the tissue, the crystals may or may not induce inflammation and disease.

To conclude, we reiterate that the proposed hypothesis has already been tested in its basic sense. The published experimental results show that (1) formation of CBDs in liposomal membranes precedes formation of Chol crystals (e.g., [17, 18, 20, 30, 31]), and (2) Chol crystals can be formed from membrane Chol (without the external addition of Chol) when the Chol solubility threshold in the membrane is decreased, for example, by lipid peroxidation [21–23]. Based on the saturation recovery EPR technique, we are planning to design an experimental procedure to quantitatively estimate the average amount of Chol in a CBD at the saturation threshold in bilayers made of polyunsaturated phospholipids (before peroxidation). It is reasonable to assume that when the system is in equilibrium, the sizes of CBDs in the bilayer are similar to each other. These polyunsaturated PC-Chol bilayers will be subjected to peroxidation, which should decrease the Chol solubility threshold and induce the formation of Chol crystals. The sizes of the individual Chol crystals will be estimated using electron microscopy or another method. If the average size of the Chol crystal is similar to the estimated size of a CBD, then we will have achieved convincing proof of our hypothesis.
